# Cochlear Implant Complications and Outcomes: National Trends of the MAUDE Database From 2016 to 2024

**DOI:** 10.1002/ohn.70297

**Published:** 2026-05-22

**Authors:** Daniel R. S. Habib, Anthony E. Bishay, Michael W. S. Habib, Ankita Patro, Alexander J. Langerman, Aaron C. Moberly, David S. Haynes, Kareem O. Tawfik

**Affiliations:** ^1^ Vanderbilt University School of Medicine Nashville Tennessee USA; ^2^ Department of Computer Science Duke University Durham North Carolina USA; ^3^ Department of Otolaryngology–Head and Neck Surgery Vanderbilt University Medical Center Nashville Tennessee USA

**Keywords:** cochlear implant, medical device failure, otology, treatment outcome

## Abstract

**Objective:**

As cochlear implant (CI) use expands, complications have become a growing concern. While prior studies analyzed CI failures, few have comprehensively assessed national complication temporal patterns and clinical outcomes. This study evaluates patient and device complication patterns and outcomes from 2016 to 2024.

**Study Design:**

Cross‐sectional.

**Setting:**

Multi‐institution database.

**Methods:**

Adverse events related to CIs from 2016 to 2024 were extracted from the US Food and Drug Administration (FDA) Manufacturer and User Facility Device Experience (MAUDE) database using CI‐specific product codes. Complications and outcomes were categorized into themes. Poisson and quasi‐Poisson regressions assessed trends in raw counts, and Cochran‐Armitage tests evaluated proportional changes of specific complications and outcomes to total counts before (2016‐2020) and after (2020‐2024) a major voluntary field corrective action.

**Results:**

From 2016 to 2024, 34,426 patient complications, 33,487 device complications, and 26,377 clinical outcomes were reported. Between 2016 and 2020, all domains exhibited rising incidence and shifting complication proportions. From 2020 to 2024, overall complication counts plateaued or declined; however, specific issues such as healing/skin, infection/inflammation, and device fit/malposition/detachment continued to increase in proportion to the total number of complications. Hospitalizations and life‐threatening outcomes also rose in proportion during this period.

**Conclusion:**

Although overall CI complication reporting has stabilized post‐2020, several complication subtypes and adverse outcomes continue to rise. These findings highlight evolving safety profiles of CIs and underscore the need for targeted device and surgical refinements as well as robust post‐market surveillance to guide safer innovation.

Hearing impairment affects a significant portion of the global population, with an estimated 1.57 billion individuals experiencing some degree of hearing loss.^1^ In the United States alone, 22.2% of the population is affected.^2^ As the prevalence of hearing loss has risen, so too has the adoption of cochlear implantation: the number of total cochlear implants (CIs) in the United States exceeds 180,000, and the annual incidence continues to increase.^3^, ^4^ While CIs have become more common, concerns remain regarding their associated risks.^5^, ^6^ Single‐center studies report implantation‐related complication rates ranging from 9.2% to 19.9%,^7^, ^8^, ^9^, ^10^ but these findings are largely institution‐dependent. Consequently, these studies may not accurately reflect true national complication rates. A clearer understanding of these risks is necessary not only to improve patient counseling and informed consent but also to refine device design and surgical approaches.

The Manufacturer and User Facility Device Experience (MAUDE) database, established by the US Food and Drug Administration (FDA), requires manufacturers, importers, and device user facilities to report complications resulting in death or serious injury.^11^ Given the role of the MAUDE database in tracking device‐related adverse events, several studies have utilized this database to investigate CI failures. For instance, temporal patterns in CI complications before 1998, compared with those in 2002, showed a decline in device failures but a rise in infection rates over time.^12^ From 2000 to 2010, an increasing proportion of adverse events were due to unknown causes.^5^ More recently, adverse events between 2010 and 2020 differed across 3 manufacturers in infection rates, cerebrospinal fluid leaks, extrusion risk, and facial nerve stimulation.^6^

Despite these findings, prior work has primarily focused on device‐related failures or a subset of complications, rather than providing a comprehensive national analysis.^5^, ^6^, ^12^, ^13^ No study has covered national temporal patterns in complications in the last 4 years or comprehensive clinical outcomes during any time period. Despite some single‐institution reports,^14^ national temporal patterns before and after the 2020 Advanced Bionics (AB) major voluntary field corrective action (VFCA) have not been assessed. Therefore, this cross‐sectional multi‐institution database study aims to bridge this gap by examining patient and device complications as well as clinical outcomes associated with CIs from 2016 to 2024 with a particular emphasis on temporal patterns before and after a major VFCA.

## Methods

### Data Collection

The FDA's publicly available MAUDE database contains several file types about medical devices.^11^ Patient files contain information about the patient such as basic demographics and outcomes (ie, required intervention, hospitalization, life‐threatening event/disability/death). Device files contain information about the device such as manufacturer and classification code. Patient problem files contain information about issues/complications related to the patient treated with the medical device such as infection or bleeding. Device problem files contain information about issues/complications with the device itself such as unexpected output or electrical failure. Problems were reported as they were labelled in the MAUDE database. Patient, device, patient problem, and device problem files from 2016 through 2024 were downloaded from the FDA's publicly available MAUDE database.^11^ Device file entries containing product codes for “cochlear implant” (MCM) and “cochlear implant with combined electrical stimulation and acoustic amplification” (PGQ) were cross‐referenced by report ID to extract the corresponding patient, patient problem, and device problem entries. Variables of interest from these files were extracted: patient outcomes, patient complications, and device complications, respectively. Multiple outcomes and complications may be reported within a single MAUDE report; for example, a patient may experience both infection and device extrusion, leading to hospitalization and a required intervention. Accordingly, analyses of complications were conducted at the event level, whereas analyses of clinical outcomes were conducted at the patient level, consistent with the structure and intended use of the MAUDE database. The database also contains overlap in how patient and device complications are coded (eg, “unexpected result” may be reported as both a patient and device complication). This study does not involve human subjects and does not require institutional review board (IRB) approval since all data is de‐identified and made publicly available by the FDA.^11^

### Analysis

A total of 317 complications were categorized into 18 themes derived from the coded MAUDE complication labels (Supplemental Tables S1–2, available online). These themes were developed inductively to consolidate semantically similar patient‐ and device‐reported complications into analytically tractable categories while preserving clinical relevance. Two researchers independently categorized the possible complication entries and met to discuss discrepancies. Patient and device complications as well as clinical outcomes were plotted as the number and percent of the total number of complications/outcomes by year to account for differences in the number of CIs implanted. Poisson regression models were performed to assess trends in complication counts across years, and quasi‐Poisson models were applied when overdispersion was detected (dispersion ratio >1.5), reporting incidence rate ratios (IRRs) with 95% confidence intervals (CIs). Cochran‐Armitage trend tests were performed to assess the presence and direction of linear trends in proportions across years. Specifically, Poisson regression models and Cochran‐Armitage trend tests were performed for 2016 to 2020 as well as 2020 to 2024 to assess differences in absolute count and proportions, respectively, during the time periods before and after a major VFCA that occurred in 2020. We performed all statistical analyses using R statistical software version 4.3.3. Significance was set a priori at *P* < .05. This study was conducted in accordance with the Strengthening the Reporting of Observational Studies in Epidemiology (STROBE) reporting guidelines (Supplemental Table S3, available online).

## Results

### Patient Complications

**Figure 1:**
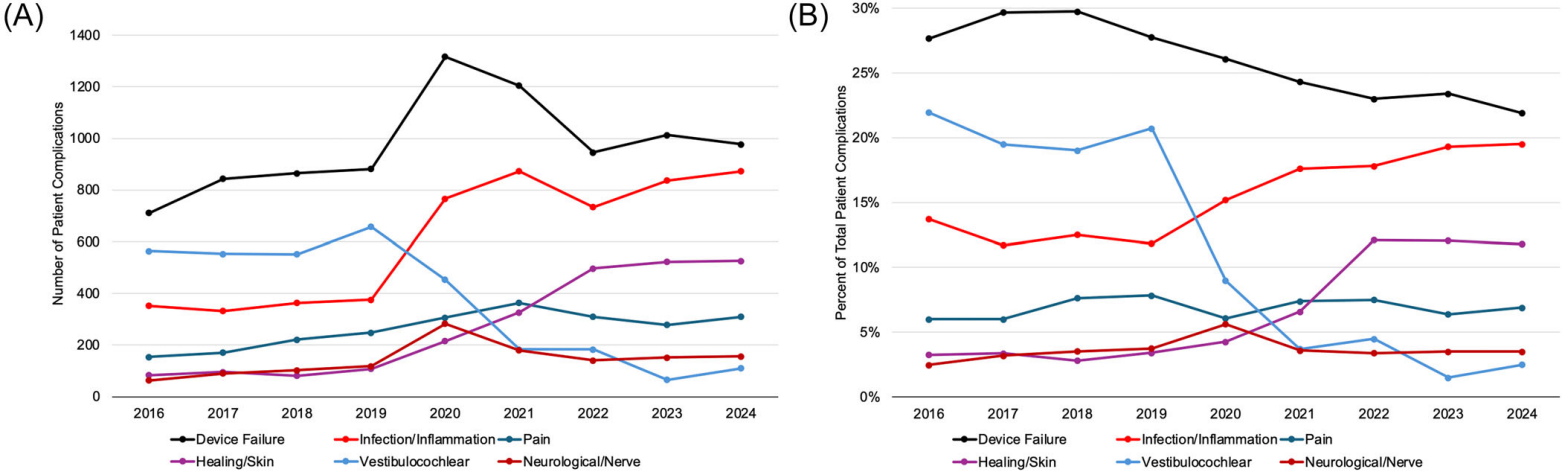
Number (A) and percent (B) of top patient complication themes from 2016 to 2024.

There was a total of 34,426 patient complications reported during the study period. From 2016 to 2020, total patient complications increased significantly from 2575 to 5050 cases (Figure 1 and Table 1). Patient complications with significantly rising incidence included device failure/unexpected result, fluid buildup/discharge, and bleeding complications. There were significant rises in the relative proportion of idiopathic/other, infection/inflammation, healing/skin, neurological/nerve, fluid buildup/discharge, and bleeding complications. There were significant dips in the proportion of device failure/unexpected result, vestibulocochlear, and erosion/perforation complications.

**Table 1:** Patient Complication Themes by Year

Patient complication	2016	2017	2018	2019	2020	2021	2022	2023	2024	2016‐20 IRR (95% CI), *P* value	2020‐24 IRR (95% CI), *P* value	2016‐20 *Z* Score, *P* value	2020‐24 Z Score, *P* Value
Idiopathic/other	628 (24.39%)	730 (25.67%)	695 (23.90%)	770 (24.23%)	1642 (32.51%)	1760 (35.56%)	1276 (31.01%)	1433 (33.04%)	1457 (32.60%)	1.54 (1.05‐2.26), *P* = .112	0.81 (0.62‐1.05), *P* = .210	***Z*** = **7.76, *P*** < .001***	*Z* = −1.09, *P* = .278
Device failure/unexpected result	712 (27.65%)	844 (29.68%)	865 (29.75%)	882 (27.75%)	1317 (26.08%)	1205 (24.34%)	946 (22.99%)	1013 (23.36%)	977 (21.86%)	**1.15 (1.06‐1.24), *P*** = **.042** *	0.92 (0.88‐0.97), *P* = .058	***Z*** = **−2.71, p** = **.007** **	***Z*** = **−4.85, *P*** < .001***
Infection/inflammation	353 (13.71%)	333 (11.71%)	364 (12.52%)	376 (11.83%)	767 (15.19%)	873 (17.64%)	734 (17.84%)	837 (19.30%)	873 (19.53%)	0.80 (0.46‐1.40), *P* = .492	0.95 (0.61‐1.49), *P* = .841	***Z*** = **2.70, *P*** = **.007** **	***Z*** = **5.92, *P*** < .001***
Pain	155 (6.02%)	171 (6.01%)	222 (7.63%)	249 (7.84%)	307 (6.08%)	364 (7.35%)	310 (7.53%)	279 (6.43%)	310 (6.94%)	3.01 (1.08‐8.39), *P* = .126	1.00 (0.52‐1.91), *P* = .996	*Z* = 0.64, *P* = .525	*Z* = 0.78, *P* = .438
Healing/skin	84 (3.26%)	96 (3.38%)	82 (2.82%)	109 (3.43%)	216 (4.28%)	327 (6.61%)	498 (12.10%)	523 (12.06%)	527 (11.79%)	1.27 (1.07‐1.52), *P* = .075	**1.22 (1.08‐1.38), *P*** = **.047** *	***Z*** = **2.60, *P*** = **.009** **	***Z*** = **15.66, *P*** < .001***
Vestibulocochlear	565 (21.94%)	554 (19.48%)	553 (19.02%)	658 (20.70%)	454 (8.99%)	184 (3.72%)	184 (4.47%)	66 (1.52%)	111 (2.48%)	1.27 (1.07‐1.51), *P* = .076	0.95 (0.89‐1.03), *P* = .299	***Z*** = **−14.42, *P*** < .001***	***Z*** = **−16.38, *P*** < .001***
Neurological/nerve	64 (2.49%)	91 (3.20%)	103 (3.54%)	119 (3.74%)	284 (5.62%)	180 (3.64%)	141 (3.43%)	152 (3.50%)	157 (3.51%)	1.22 (1.03‐1.45), *P* = .102	1.02 (0.97‐1.07), *P* = .461	***Z*** = **7.03, *P*** < .001***	***Z*** = **−4.97, *P*** < .001***
Fluid buildup/discharge	3 (0.12%)	0 (0%)	1 (0.03%)	7 (0.22%)	33 (0.65%)	29 (0.59%)	133 (2.27%)	24 (0.55%)	36 (0.81%)	**1.46 (1.23‐1.73), *P*** = **.023** *	0.86 (0.75‐0.98), *P* = .102	***Z*** = **5.51, *P*** < .001***	*Z* = 0.91, *P* = .363
Bleeding	6 (0.23%)	6 (0.21%)	7 (0.24%)	5 (0.16%)	27 (0.53%)	20 (0.40%)	9 (0.22%)	8 (0.18%)	16 (0.36%)	**1.19 (1.14‐1.24), *P*** < .001***	0.98 (0.91‐1.04), *P* = .497	***Z*** = **2.39, *P*** = **.017** *	***Z*** = **−2.17, *P*** = **.030** *
Erosion/perforation	5 (0.19%)	19 (0.67%)	16 (0.55%)	3 (0.09%)	3 (0.06%)	8 (0.16%)	2 (0.05%)	2 (0.05%)	5 (0.11%)	0.98 (0.89‐1.07), *P* = .680	0.65 (0.51‐0.82), *P* = .038	***Z*** = **−3.53, *P*** < .001***	*Z* = −0.07, *P* = .944
Total	2575 (100%)	2844 (100%)	2908 (100%)	3178 (100%)	5050 (100%)	4950 (100%)	4115 (100%)	4337 (100%)	4469 (100%)	**1.18 (1.07‐1.30), *P*** = **.048** *	0.96 (0.92‐1.01), *P* = .186	NA	NA

Multiple patient complications were possible for each patient. Percentages are reported with respect to total number of complications, not patients. Incidence rate ratios greater than one indicate increasing trends in complication counts across years while incidence rate ratios less than one indicate decreasing trends. Positive *Z* scores indicate increasing linear trends in proportions across years while negative *Z* scores indicate decreasing trends.

Abbreviations: CI, confidence interval; IRR, incidence rate ratio; NA, not applicable.

* *P* < .05.

** *P* < .01.

***P < .001.

From 2020 to 2024, total patient complications decreased from 5050 to 4469 cases without statistical significance (Figure 1 and Table 1). Only healing/skin complications exhibited a significantly rising incidence. There were significant rises in the relative proportion of infection/inflammation and healing/skin complications. There were significant dips in the proportion of device failure/unexpected result, vestibulocochlear, neurological/nerve, and bleeding complications.

### Device Complications

**Figure 2:**
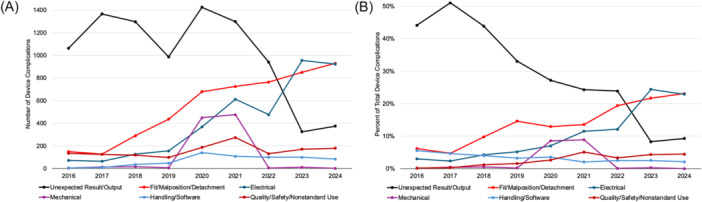
Number (A) and percent (B) of top device complication themes from 2016 to 2024.

There was a total of 33,487 device complications reported during the study period. From 2016 to 2020, total device complications significantly increased from 2413 to 5242 cases (Figure 2 and Table 2). Device complications with significantly rising incidence included idiopathic/other, fit/malposition/detachment, electrical, and handling/software issues. There were significant rises in the relative proportion of fit/malposition/detachment, electrical, mechanical, and quality/safety/nonstandard use complications. In contrast, there were significant dips in the proportion of unexpected result/output and handling/software complications.

**Table 2:** Device Complication Themes by Year

Device complication	2016	2017	2018	2019	2020	2021	2022	2023	2024	2016‐20 IRR (95% CI), *P* value	2020‐24 IRR (95% CI), *P* value	2016‐20 *Z* score, *P* value	2020‐24 *Z* score, *P* value
Idiopathic/other	974 (40.36%)	970 (36.25%)	1074 (36.32%)	1255 (42.04%)	1979 (37.75%)	1848 (34.56%)	1513 (38.50%)	1494 (38.18%)	1524 (37.87%)	**1.20 (1.09‐1.33), *P*** = **.037** *	**0.93 (0.89‐0.97), *P*** = **.041** *	*Z* = 0.03, *P* = .978	*Z* = 1.71, *P* = .088
Unexpected result/output	1064 (44.09%)	1366 (51.05%)	1297 (43.86%)	986 (33.03%)	1425 (27.18%)	1298 (24.28%)	940 (23.92%)	324 (8.28%)	374 (9.29%)	1.03 (0.92‐1.14), *P* = .645	**0.69 (0.58‐0.82), *P*** = **.024** *	***Z*** = **−21.33, *P*** < **.001**	***Z*** = **−27.50, *P*** < .001***
Fit/malposition/detachment	149 (6.17%)	126 (4.71%)	289 (9.77%)	436 (14.61%)	679 (12.95%)	725 (13.56%)	764 (19.44%)	850 (21.72%)	930 (23.11%)	**1.55 (1.39‐1.74), *P*** = **.005** **	**1.08 (1.06‐1.11), *P*** < .001***	***Z*** = **13.43, *P*** < .001***	***Z*** = **16.07, *P*** < .001***
Electrical	72 (2.98%)	63 (2.35%)	126 (4.26%)	155 (5.19%)	368 (7.02%)	612 (11.45%)	476 (12.11%)	956 (24.43%)	923 (22.94%)	**1.61 (1.34‐1.93), *P*** = **.014** *	1.25 (1.08‐1.44), *P* = .057	***Z*** = **10.03, *P*** < .001***	***Z*** = **26.70, *P*** < .001***
Mechanical	3 (0.12%)	12 (0.45%)	16 (0.54%)	6 (0.20%)	448 (8.55%)	475 (8.88%)	4 (0.10%)	10 (0.26%)	1 (0.02%)	6.63 (1.49‐29.38), *P* = .089	0.37 (0.15‐0.89), *P* = .113	***Z*** = **22.49, *P*** < .001***	***Z*** = **−28.01, *P*** < .001***
Quality/safety/nonstandard use	4 (0.17%)	8 (0.30%)	35 (1.18%)	47 (1.57%)	139 (2.65%)	273 (5.11%)	130 (3.31%)	170 (4.34%)	179 (4.45%)	1.06 (0.90‐1.24), *P* = .534	**0.83 (0.74‐0.93), *P*** = **.047** *	***Z*** = **10.19, *P*** < .001***	***Z*** = **3.13, *P*** = **.002** **
Handling/software	134 (5.55%)	125 (4.67%)	118 (3.99%)	97 (3.25%)	186 (3.55%)	108 (2.02%)	99 (2.52%)	99 (2.53%)	83 (2.06%)	**2.34 (1.91‐2.87), *P*** = **.004** **	0.99 (0.79‐1.24), *P* = .918	***Z*** = **−4.56, *P*** < .001***	***Z*** = **−3.43,** *P* = .001**
Procedural/maintenance	13 (0.54%)	6 (0.22%)	2 (0.07%)	3 (0.10%)	18 (0.34%)	8 (0.15%)	4 (0.10%)	10 (0.26%)	10 (0.25%)	1.09 (0.62‐1.92), *P* = .792	0.87 (0.63‐1.19), *P* = .443	*Z* = −0.90, *P* = .370	*Z* = −0.53, *P* = .593
Total	2413 (100%)	2676 (100%)	2957 (100%)	2985 (100%)	5242 (100%)	5347 (100%)	3930 (100%)	3913 (100%)	4024 (100%)	**1.20 (1.08‐1.35), *P*** = **.049** *	0.92 (0.86‐0.98), *P* = .075	NA	NA

Multiple device complications were possible for each patient. Percentages are reported with respect to total number of complications, not patients. Incidence rate ratios greater than 1 indicate increasing trends in complication counts across years while incidence rate ratios less than one indicate decreasing trends. Positive *Z* scores indicate increasing linear trends in proportions across years while negative *Z* scores indicate decreasing trends.

Abbreviations: CI, confidence interval; IRR, incidence rate ratio; NA, not applicable.

* *P* < .05.

** *P* < .01.

***P < .001.

From 2020 to 2024, total device complications decreased from 5242 to 4024 cases with a nonsignificant trend toward lower incidence (Figure 2 and Table 2). The only device complication type with significantly rising incidence included fit/malposition/detachment issues. Device complications with significantly decreasing incidence included idiopathic/other, unexpected result/output, and quality/safety/nonstandard use issues. There were significant rises in the relative proportion of fit/malposition/detachment and electrical complications. In contrast, there were significant dips in the proportion of unexpected result/output, mechanical, and handling/software complications.

In 2020, there was a large spike in reported device complications regarding AB CIs (Supplemental Figure S1, available online). Less than two percent of total device complications were related to AB CIs from 2016 to 2019. In 2020, the number of device complications increased from 2985 to 5242, with 42% being related to AB CIs. In 2021, 32% of the 5347 device complications were related to AB CIs. From 2022 to 2024, AB CI device complications again constituted less than two percent of all reported device complications.

### Clinical Outcomes

**Figure 3:**
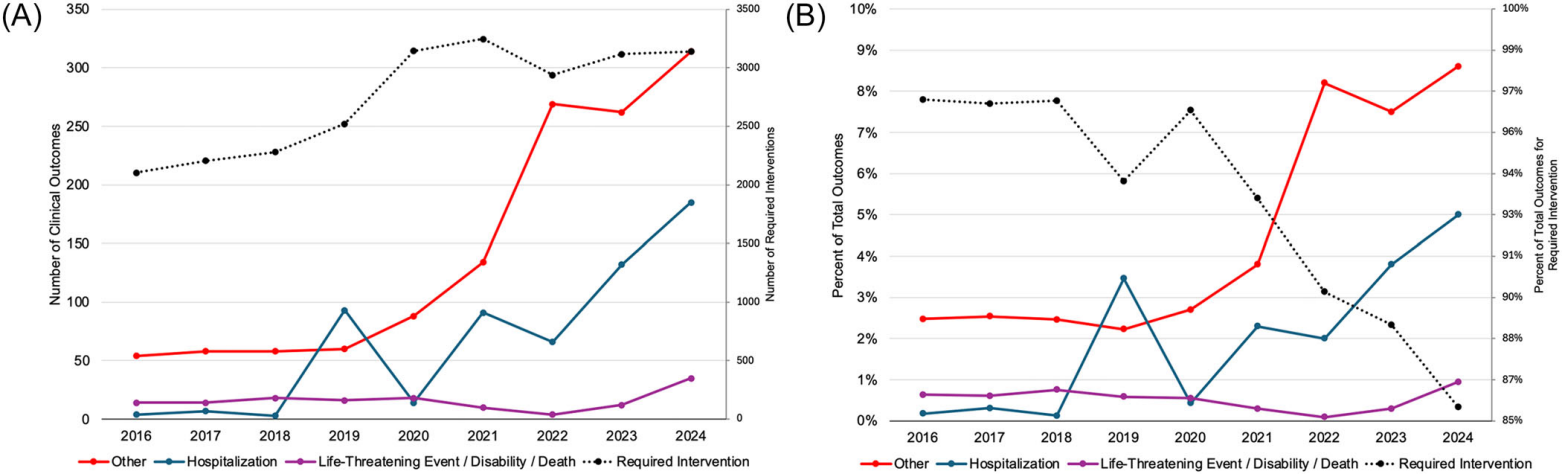
Number (A) and percent (B) of clinical outcomes from 2016 to 2024.

There was a total of 26,377 clinical outcomes reported during the study period. From 2016 to 2020, total reported clinical outcomes significantly increased from 2176 to 3265 cases (Figure 3 and Table 3). Clinical outcomes with significantly rising incidence included required intervention and “other” outcomes. There was a significant rise in the relative proportion of hospitalizations but a significant dip in the proportion of required interventions.

**Table 3:** Clinical Outcomes of Complications by Year

Outcome	2016	2017	2018	2019	2020	2021	2022	2023	2024	2016‐20 IRR (95% CI), *P* value	2020‐24 IRR (95% CI), *P* value	2016‐20 *Z* score, *P* value	2020‐24 *Z* score, *P* value
Required intervention	2104 (96.69%)	2204 (96.54%)	2280 (96.65%)	2521 (93.72%)	3145 (96.32%)	3246 (93.09%)	2939 (89.66%)	3117 (88.48%)	3139 (85.46%)	**1.10 (1.06‐1.15), *P*** = **.022** *	1.00 (0.97‐1.02), *P* = .747	***Z*** = **−2.53, *P*** = **.011** *	***Z*** = **−5.55, *P*** < .001***
Other	54 (2.48%)	58 (2.54%)	58 (2.46%)	60 (2.23%)	88 (2.70%)	134 (3.84%)	269 (8.21%)	262 (7.44%)	314 (8.55%)	**1.12 (1.03‐1.21), *P*** = **.006** **	**1.32 (1.14‐1.55), *P*** = **.038** *	*Z* = 0.22, *P* = .823	***Z*** = **12.85, *P*** < .001***
Hospitalization	4 (0.18%)	7 (0.31%)	3 (0.13%)	93 (3.46%)	14 (0.43%)	97 (2.78%)	66 (2.01%)	132 (3.75%)	185 (5.04%)	1.62 (0.55‐4.73), *P* = .445	1.50 (1.14‐1.99), *P* = .064	***Z*** = **5.20,** *P* < .001***	***Z*** = **12.12, *P*** < .001***
Life‐threatening event/disability/death	14 (0.64%)	14 (0.61%)	18 (0.76%)	16 (0.59%)	18 (0.55%)	10 (0.29%)	4 (0.09%)	12 (0.34%)	35 (0.95%)	1.06 (0.91‐1.24), *P* = .430	1.26 (0.80‐1.99), *P* = .390	*Z* = −0.48, p = .628	***Z*** = **2.84, *P*** = **.005** **
Total	2176 (100%)	2283 (100%)	2359 (100%)	2690 (100%)	3265 (100%)	3487 (100%)	3278 (100%)	3523 (100%)	3316 (100%)	**1.11 (1.06‐1.15), *P*** = **.015** *	1.00 (0.98‐1.03), *P* = .773	NA	NA

Multiple outcomes were possible for each patient. Percentages are reported with respect to total number of outcomes, not patients. Incidence rate ratios greater than one indicate increasing trends in complication counts across years while incidence rate ratios less than one indicate decreasing trends. Positive *Z* scores indicate increasing linear trends in proportions across years while negative *Z* scores indicate decreasing trends.

Abbreviations: CI, confidence interval; IRR, incidence rate ratio; NA, not applicable.

* *P* < .05.

** *P* < .01.

***P < .001.

From 2020 to 2024, total reported clinical outcomes decreased slightly from 3265 to 3316 cases without a significant trend (Figure 3 and Table 3). Only “other” outcomes exhibited a significantly rising incidence. There were significant rises in the relative proportion of “other,” hospitalization, and life‐threatening/disability/death outcomes. In contrast, there was a significant dip in the proportion of required interventions.

## Discussion

During the study period from 2016 to 2024, a total of 34,426 patient complications, 33,487 device complications, and 26,377 clinical outcomes were reported. From 2016 to 2020, all three domains exhibited significant increases in total reports, with notable rises in device failure, fluid buildup/discharge, bleeding, and required interventions. This period also saw shifting proportions in complication types, including increases in idiopathic, infection/inflammation, and healing/skin‐related issues. In contrast, from 2020 to 2024, total counts across all categories plateaued or declined without significant trends, though certain subtypes—such as healing/skin complications and fit/malposition/detachment issues—continued to rise in both absolute and proportional terms. These findings suggest that while the overall reporting burden has stabilized in recent years, specific complication types remain on the rise, warranting targeted investigation and potential quality improvement efforts.

CIs have transformed the management of severe‐to‐profound hearing loss, but ongoing surveillance is essential to assess their safety and effectiveness. While hospitalizations and life‐threatening events/disability/death increased, the proportion of interventions did not. Given their classification as high‐risk (Class III) medical devices, CIs undergo stricter regulatory oversight than devices of lower classification through the premarket approval (PMA) process to ensure safety and effectiveness.^15^, ^16^ In line with this sentiment, a CI was voluntarily withdrawn from the market due to hearing performance degradation concerns in 2020, highlighting the importance of ongoing postmarket surveillance.^17^ Despite a nonsignificant trend toward decreased device complications after this VFCA, there remained a high incidence of adverse clinical outcomes. A discrepancy is expected, as our patient population spans all age groups, and importantly, the current study shows a shift in reporting temporal patterns, particularly in postmarket surveillance categories such as death, disability, and hospitalizations, underscoring the need for continued monitoring and reporting of adverse events.

There has been a clear shift in reported CI device and patient complications. While the proportion of some device‐related complications such as mechanical failures has significantly declined, others such as fit/malposition/detachment issues have become increasingly prevalent. The significant decline in device failures may be attributable to improvements in both manufacturing and surgical techniques,^18^, ^19^ although direct comparisons are challenging due to the lack of standardized definitions in the MAUDE database.^20^ For example, a single‐institution study by Layfield et al^21^ encompassing a 10‐year period showed that the device failure rate was only 5.9% across 31 patients. These findings suggest that as CI technology has evolved, fundamental hardware reliability has improved, but fit/malposition/detachment, quality/safety/nonstandard use, and electrical issues not rising to the level of device failure still require attention. Clinicians and industry executives can leverage these temporal patterns to guide data‐driven quality improvement initiatives.

While prior MAUDE‐based studies analyzed individual line‐item complications, the current approach of grouping related issues into broader categories may provide a more comprehensive view of complication temporal patterns, potentially revealing underlying relationships between different types of complications that could inform device design and patient care. For example, Causon et al^5^ reported an infection rate of 0.71% from 2010 to 2020. In contrast, the current study combined infectious and inflammatory etiologies to better capture the full spectrum of these complications, an approach particularly relevant given the exceptionally low overall complication rates in cochlear implantation. Future research should adopt similar methods to enhance comparability and more accurately track temporal patterns in device performance among a vast, otherwise undigestible dataset.

### Limitations

The current study has several limitations that should be acknowledged. First, the retrospective nature of this study inherently limits the ability to establish causation, as the data rely on reported events rather than systematic prospective collection. Temporal patterns may indicate increased reporting, emerging device‐related risks, or a growing patient population receiving implants––underlying causes that cannot be fully teased apart in this analysis. Additionally, while statistically significant trends were identified using Poisson regression and Cochran‐Armitage tests, the clinical significance of these trends remains uncertain. Second, the MAUDE database is subject to reporting bias due to its voluntary nature for certain complications: 96.6% of MAUDE reports originate from manufacturers,^22^ with minimal input from consumers, patients, and clinicians.^23^ Minor complications such as device handling issues, mild skin irritation, or transient discomfort are likely underreported, whereas severe adverse events, including device failure, infections, or surgical complications, are overrepresented due to mandatory reporting requirements. This imbalance may skew the perceived complication rates and limit generalizability. Third, the MAUDE database lacks standardized, precisely defined complication categories, and individual reports may include overlapping or ambiguously coded events across patient and device problem files. To address this, we grouped reported complications into discrete thematic categories to facilitate analysis; however, these themes were investigator‐derived and may not fully align with the original classifications intended at the time of data entry. As a result, some events may have been misclassified or omitted, and direct comparisons with prior studies using different classification schemes may be limited. This represents an inherent limitation of analyses that rely on post hoc categorization of large adverse‐event databases, such as MAUDE. Fourth, complication and outcome trends in this study are reported relative to the total number of events reported in each year rather than normalized to an external procedural denominator (eg, the annual number of CI surgeries). Although this approach allows for comparison of relative changes in reported complication patterns over time, it does not control for increases in cochlear implantation volume, shifts in patient eligibility (eg, changes in Medicare coverage), or changes in awareness and compliance with MAUDE reporting requirements. Accurately standardizing for these factors—or benchmarking cochlear implantation trends against surgeries for implanting other medical devices—would require reliable, contemporaneous procedural volume data and harmonized reporting practices across device classes, which are not available in MAUDE and fall outside the scope of this database. Moreover, different device classes are subject to distinct regulatory environments, reimbursement policies, patient populations, and clinical indications, such that temporal trends in one device category may be driven by external factors that are not comparable across devices. As a result, cross‐device comparisons may introduce additional confounding rather than serve as an appropriate control. Future studies integrating MAUDE data with national procedural registries, payer claims, or institutional datasets may enable denominator‐adjusted and policy‐sensitive analyses. Finally, the dataset does not include comprehensive clinical details, such as patient comorbidities, variations in surgical technique, or long‐term functional outcomes, which are critical for contextualizing complications. Although certain device manufacturer and demographic variables—most notably patient age—are available within MAUDE patient files, these fields are missing or inconsistently reported in most records, limiting their analytic utility. This incompleteness in patient‐level characteristics precluded reliable stratified or multivariable analyses correlating complication themes with patient demographics. Consequently, thematic categories were used to characterize population‐level reporting patterns rather than individual‐level risk factors. Despite known issues with MAUDE,^24^ no other dataset contains a similar magnitude of device complication data. Future studies integrating MAUDE with clinical registries or institutional datasets may allow more granular, risk‐adjusted analyses of cochlear implant complications.

### Implications and Future Directions

Our findings provide actionable insights for patients, providers, payers, and policymakers as cochlear implantation continues to grow in the US market. For patients, recognizing that certain complication types (eg, infection, healing/skin issues, and fit/malposition/detachment) persist underscores the importance of comprehensive preoperative counseling that reflects not only historical risks but contemporary, data‐driven trends. Providers should prioritize preoperative risk stratification and perioperative protocols that specifically target these rising complication categories, especially as hardware failures have declined but soft tissue and anatomical challenges persist. Surgeons may also consider adopting or developing more advanced imaging, intraoperative guidance, and postoperative monitoring techniques tailored to anatomical fit and healing dynamics. From a payer perspective, our data can inform value‐based reimbursement strategies that incentivize centers achieving lower complication rates in these specific categories, rather than broadly focusing on all‐cause complications. Finally, for policymakers and regulatory agencies, these trends reaffirm the importance of strengthening post‐market surveillance mechanisms. The 2020 VFCA was a critical moment, but sustained monitoring—potentially through mandated reporting for a broader range of complications—could preempt future safety issues. Moreover, the FDA and manufacturers may use these data to guide targeted post‐approval studies focused on emerging complication types, rather than defaulting to general device safety assessments. Using national databases like MAUDE may provide insights to supplement individual studies or meta‐analyses to assess CI outcomes.^25^ Future national surveillance efforts could benefit from integrating MAUDE data with clinical registries that capture patient‐level variables and long‐term outcomes, providing a more holistic framework to advance both device safety and patient care.

## Conclusion

From 2016 to 2020, reported patient, device, and clinical outcome complications significantly increased, followed by stabilization or decline from 2020 to 2024. Despite this plateau, certain complications such as healing/skin issues and fit/malposition/detachment issues continued to rise, highlighting evolving areas of risk. These patterns underscore the need for targeted device design improvements, enhanced surveillance, and proactive patient safety interventions.

## Author Contributions

**Daniel R. S. Habib**, study design, data collection, data analysis, manuscript writing, manuscript revision; **Anthony E. Bishay**, study design, data analysis, manuscript writing, manuscript revision; **Michael W. S. Habib**, data collection, data analysis, manuscript revision; **Ankita Patro**, study design, manuscript revision; **Alexander J. Langerman**, study design, manuscript revision; **Aaron C. Moberly**, study design, manuscript revision; **David S. Haynes**, study design, manuscript revision; **Kareem O. Tawfik**, study design, manuscript revision.

## Disclosures

### Competing interests

None.

### Funding source

None.

## Supporting information


**Supplemental Table 1**. Patient Complications by Theme.
**Supplemental Table 2**. Device Complications by Theme.
**Supplemental Table 3**. STROBE Statement—checklist of items that should be included in reports of observational studies.
**Supplemental Figure 1.** Number and Percent of Advanced Bionics Device Complications from 2016 to 2024.
