# Supplementary material for: Cochlear Implant Complications and Outcomes: National Trends of the MAUDE Database From 2016 to 2024
Source: Otolaryngol Head Neck Surg. 2026 May 22;175(2):444–52. doi: 10.1002/ohn.70297 (PMC13417938; doi:10.1002/ohn.70297)
Supplement: Supplementary file 1 — Supplemental Table 1. Patient Complications by Theme. Supplemental Table 2. Device Complications by Theme. Supplemental Table 3. STROBE Statement—checklist of items that should be included in reports of observational studies. Supplemental Figure 1. Number and Percent of Advanced Bionics Device Complications from 2016 to 2024. [file OHN-175-444-s001.docx]

**Supplemental Table 1**. Patient Complications by Theme

| **Patient Complications by Theme** |
| --- |
| **Bleeding** |
| Blood Loss |
| Bruise/Contusion |
| Hematoma |
| Hemorrhage/Blood Loss/Bleeding |
| **Device Failure/Unexpected Result** |
| Failure of Implant |
| Device Overstimulation of Tissue |
| Reaction to Medicinal Component of Device |
| Therapeutic Effects\| Unexpected |
| Therapeutic Response\| Decreased |
| **Erosion/Perforation** |
| Device Embedded in Tissue or Plaque |
| Erosion |
| Perforation |
| Pocket Erosion |
| Rupture |
| **Fluid Buildup/Discharge** |
| Discharge |
| Fluid Discharge |
| Seroma |
| **Healing/Skin** |
| Abrasion |
| Blister |
| Burn(s) |
| Burn\| Thermal |
| Contact Dermatitis |
| Eczema |
| Erythema |
| Fibrosis |
| Granuloma |
| Hyperemia |
| Hyperplasia |
| Impaired Healing |
| Irritation |
| Laceration(s) |
| Localized Skin Lesion |
| Necrosis |
| Necrosis of Flap Tissue |
| Phototoxicity |
| Pressure Sores |
| Rash |
| Scar Tissue |
| Skin Discoloration |
| Skin Disorders |
| Skin Erosion |
| Skin Inflammation |
| Skin Irritation |
| Skin Tears |
| Superficial (First Degree) Burn |
| Tissue Breakdown |
| Tissue Damage |
| Ulcer |
| Ulceration |
| Wound Dehiscence |
| **Idiopathic/Other** |
| Anxiety/Fatigue/Emotional Changes |
| Appropriate Term / Code Not Available |
| Arrhythmia |
| Cancer/Hair Loss/Solid Tumor |
| Complaint\| Ill-Defined |
| Death |
| Exposure to Body Fluids |
| Extraskeletal Ossification |
| Failure to Anastomose |
| Foreign Body/Immunologic Reaction |
| Inadequate Osseointegration |
| Incompetent Cervix |
| Injury |
| Insufficient Information |
| Ischemia |
| Missing Value Reason |
| No Clinical Signs\| Symptoms or Conditions |
| No Code Available |
| No Consequences or Impact to Patient |
| No Information |
| No Known Impact or Consequence to Patient |
| No Patient Involvement |
| Not Applicable |
| Organ Dehiscence |
| Patient Problem/Medical Problem |
| Respiratory Distress |
| Therapeutic Response\| Increased |
| Visual Impairment |
| **Infection/Inflammation** |
| Abscess |
| Bacterial Infection |
| Cellulitis |
| Chills |
| Cyst(s) |
| Drug-Resistant Bacterial Infection |
| Ear infection |
| Edema |
| Encephalitis |
| Fever |
| Fistula |
| Fungal Infection |
| Infectious Otitis Media |
| Inflammation |
| Mastoiditis |
| Meningitis |
| Middle Ear Inflammation |
| Osteomyelitis |
| Post Operative Wound Infection |
| Purulent Discharge |
| Pyogenic Infection |
| Sepsis |
| Septic Shock |
| Shock |
| Skin Infection |
| Skin Inflammation/ Irritation |
| Staphylococcus Aureus |
| Swelling |
| Swelling/ Edema |
| Unspecified Infection |
| Viral Infection |
| Wound Infection |
| **Neurological/Nerve** |
| Burning Sensation |
| Cerebrospinal Fluid Leakage |
| Cognitive Changes |
| Coma |
| Concussion |
| Confusion/ Disorientation |
| Convulsion/Seizure |
| Decreased Sensitivity |
| Dysphasia |
| Electric Shock |
| Encephalocele |
| Facial Nerve Paralysis |
| Facial Paralysis |
| Head Injury |
| Hemorrhage\| Cerebral |
| Hemorrhagic Stroke |
| Increased Sensitivity |
| Itching Sensation |
| Muscle Spasm(s) |
| Muscle Stimulation |
| Nerve Damage |
| Numbness |
| Paralysis |
| Photophobia |
| Polydipsia |
| Sedation |
| Seizures |
| Skin Burning Sensation |
| Stroke/CVA |
| Syncope |
| Taste Disorder |
| Transmissible Spongiform Encephalopathy (TSE) |
| Twitching |
| Undesired Nerve Stimulation |
| Unspecified Nervous System Problem |
| Weakness |
| **Pain** |
| Discomfort |
| Ear Pain |
| Headache |
| Implant Pain |
| Inadequate Pain Relief |
| Intraoperative Pain |
| Neck Pain |
| Pain |
| **Vestibulocochlear** |
| Acoustic Shock |
| Ambulation or Postural Difficulties |
| Balance Problems |
| Deafness |
| Diplopia/Double Vision |
| Dizziness |
| Fall |
| Hearing Impairment |
| Hearing Loss |
| Labyrinthitis |
| Nausea |
| Partial Hearing Loss |
| Tinnitus |
| Total Hearing Loss |
| Unspecified Ear or Labyrinth Problem |
| Vertigo |
| Vomiting |

**Supplemental Table 2**. Device Complications by Theme

| **Device Complications by Theme** |
| --- |
| **Electrical** |
| Battery Problem |
| Circuit Failure |
| Defibrillation/Stimulation Problem |
| Device Stops Intermittently |
| Electrical /Electronic Property Problem |
| Electrical Overstress |
| Electrical Shorting |
| Electromagnetic Compatibility Problem |
| Electromagnetic Interference |
| Energy Output Problem |
| Failure to Deliver Shock/Stimulation |
| High impedance |
| Impedance Problem |
| Inappropriate/Inadequate Shock/Stimulation |
| Intermittent Continuity |
| Intermittent Energy Output |
| Intermittent Loss of Power |
| Intermittent Shock/Stimulation |
| Low impedance |
| **Handling/Compatibility/Software** |
| Application Program Problem |
| Application Program Version or Upgrade Problem |
| Audible Prompt/Feedback Problem |
| Communication or Transmission Problem |
| Computer Operating System Problem |
| Connection Problem |
| Device Difficult to Program or Calibrate |
| Device Handling Problem |
| Device Or Device Fragments Location Unknown |
| Device-Device Incompatibility |
| Difficult to Open or Remove Packaging Material |
| Difficult to Remove |
| Failure of Device to Self-Test |
| Failure to Read Input Signal |
| Failure to Sense |
| Intermittent Communication Failure |
| Invalid Sensing |
| Labelling\| Instructions for Use or Training Problem |
| Loose or Intermittent Connection |
| Manufacturing\| Packaging or Shipping Problem |
| Operating System Version or Upgrade Problem |
| Patient Device Interaction Problem |
| Patient-Device Incompatibility |
| Programming Issue |
| Use of Device Problem |
| **Mechanical** |
| Bent |
| Break |
| Component Missing |
| Folded |
| Fracture |
| Kinked |
| Material Deformation |
| Material Puncture/Hole |
| Material Split\| Cut or Torn |
| Material Twisted/Bent |
| Mechanical Jam |
| Mechanical Problem |
| Mechanics Altered |
| Physical Resistance |
| Physical Resistance/Sticking |
| Structural Problem |
| **Unexpected Result/Output** |
| Decreased Sensitivity |
| Device Operates Differently Than Expected |
| Improper Device Output |
| Inaccurate Delivery |
| Inappropriate Audible Prompt/Feedback |
| Inaudible or Unclear Audible Prompt/Feedback |
| Incorrect\| Inadequate or Imprecise Result or Readings |
| Increased Sensitivity |
| Lack of Effect |
| Missing Value Reason |
| No Audible Alarm |
| No Audible Prompt/Feedback |
| No Device Output |
| Noise\| Audible |
| Output below Specifications |
| Output Problem |
| Signal Artifact/Noise |
| Therapeutic or Diagnostic Output Failure |
| Unexpected Shutdown |
| Unexpected Therapeutic Results |
| **Procedural/Maintenance** |
| Difficult to Advance |
| Difficult to Insert |
| Improper or Incorrect Procedure or Method |
| Installation-Related Problem |
| Misassembly During Maintenance/Repair |
| **Quality/Safety/Nonstandard Use** |
| Biocompatibility |
| Biofilm coating in Device |
| Burst Container or Vessel |
| Chemical Spillage |
| Contamination /Decontamination Problem |
| Corroded |
| Crack |
| Defective Component |
| Defective Device |
| Device Appears to Trigger Rejection |
| Device Damaged Prior to Use |
| Device Unsafe to Use in Environment |
| Electro-Static Discharge |
| Explosion |
| Extrusion |
| Fail-Safe Problem |
| Fire |
| Flare or Flash |
| Fluid/Blood Leak |
| Leak/Splash |
| Material Fragmentation |
| Material Protrusion/Extrusion |
| Melted |
| Microbial Contamination of Device |
| Moisture or Humidity Problem |
| Nonstandard Device |
| Off-Label Use |
| Overheating of Device |
| Product Quality Problem |
| Prophylactic Removal Due to Corrective Action |
| Smoking |
| Sparking |
| Temperature Problem |
| Thermal Decomposition of Device |
| Unintended Electrical Shock |
| **Fit/Malposition/Detachment** |
| Activation\| Positioning or Separation Problem |
| Decoupling |
| Detachment of Device or Device Component |
| Device Dislodged or Dislocated |
| Device Fell |
| Device Slipped |
| Device Tipped Over |
| Disconnection |
| Expulsion |
| Fitting Problem |
| Inadequacy of Device Shape and/or Size |
| Loosening of Implant Not Related to Bone-Ingrowth |
| Malposition of Device |
| Migration |
| Migration or Expulsion of Device |
| Positioning Failure |
| Positioning Problem |
| Unintended Movement |
| **Idiopathic/Other** |
| Adverse Event Without Identified Device or Use Problem |
| Appropriate Term/Code Not Available |
| Device Inoperable |
| Device Issue |
| Device Operational Issue |
| Insufficient Information |
| No Apparent Adverse Event |
| Osseointegration Problem |

**Supplemental Table 3**. STROBE Statement—checklist of items that should be included in reports of observational studies

|  | Item No | Recommendation | Page  No |
| --- | --- | --- | --- |
| **Title and abstract** | 1 | (*a*) Indicate the study’s design with a commonly used term in the title or the abstract | 2 |
|  |  | (*b*) Provide in the abstract an informative and balanced summary of what was done and what was found | 2 |
| Introduction | | | |
| Background/rationale | 2 | Explain the scientific background and rationale for the investigation being reported | 3-4 |
| Objectives | 3 | State specific objectives, including any prespecified hypotheses | 4 |
| Methods | | | |
| Study design | 4 | Present key elements of study design early in the paper | 5-6 |
| Setting | 5 | Describe the setting, locations, and relevant dates, including periods of recruitment, exposure, follow-up, and data collection | 5-6 |
| Participants | 6 | (*a*) *Cohort study*—Give the eligibility criteria, and the sources and methods of selection of participants. Describe methods of follow-up  *Case-control study*—Give the eligibility criteria, and the sources and methods of case ascertainment and control selection. Give the rationale for the choice of cases and controls  *Cross-sectional study*—Give the eligibility criteria, and the sources and methods of selection of participants | 5-6 |
|  |  | (*b*) *Cohort study*—For matched studies, give matching criteria and number of exposed and unexposed  *Case-control study*—For matched studies, give matching criteria and the number of controls per case | NA |
| Variables | 7 | Clearly define all outcomes, exposures, predictors, potential confounders, and effect modifiers. Give diagnostic criteria, if applicable | 5-6 |
| Data sources/ measurement | 8* | For each variable of interest, give sources of data and details of methods of assessment (measurement). Describe comparability of assessment methods if there is more than one group | *5-6* |
| Bias | 9 | Describe any efforts to address potential sources of bias | NA |
| Study size | 10 | Explain how the study size was arrived at | NA |
| Quantitative variables | 11 | Explain how quantitative variables were handled in the analyses. If applicable, describe which groupings were chosen and why | NA |
| Statistical methods | 12 | (*a*) Describe all statistical methods, including those used to control for confounding | 6 |
|  |  | (*b*) Describe any methods used to examine subgroups and interactions | 6 |
|  |  | (*c*) Explain how missing data were addressed | NA |
|  |  | (*d*) *Cohort study*—If applicable, explain how loss to follow-up was addressed  *Case-control study*—If applicable, explain how matching of cases and controls was addressed  *Cross-sectional study*—If applicable, describe analytical methods taking account of sampling strategy | NA |
|  |  | (*e*) Describe any sensitivity analyses | NA |

Continued on next page

| Results | | | |
| --- | --- | --- | --- |
| Participants | 13* | (a) Report numbers of individuals at each stage of study—eg numbers potentially eligible, examined for eligibility, confirmed eligible, included in the study, completing follow-up, and analysed | 7-9 |
|  |  | (b) Give reasons for non-participation at each stage | NA |
|  |  | (c) Consider use of a flow diagram | NA |
| Descriptive data | 14* | (a) Give characteristics of study participants (eg demographic, clinical, social) and information on exposures and potential confounders | 7-9 |
|  |  | (b) Indicate number of participants with missing data for each variable of interest | NA |
|  |  | (c) *Cohort study*—Summarise follow-up time (eg, average and total amount) | NA |
| Outcome data | 15* | *Cohort study*—Report numbers of outcome events or summary measures over time | NA |
|  |  | *Case-control study—*Report numbers in each exposure category, or summary measures of exposure | NA |
|  |  | *Cross-sectional study—*Report numbers of outcome events or summary measures | 7-9 |
| Main results | 16 | (*a*) Give unadjusted estimates and, if applicable, confounder-adjusted estimates and their precision (eg, 95% confidence interval). Make clear which confounders were adjusted for and why they were included | 7-9 |
|  |  | (*b*) Report category boundaries when continuous variables were categorized | NA |
|  |  | (*c*) If relevant, consider translating estimates of relative risk into absolute risk for a meaningful time period | NA |
| Other analyses | 17 | Report other analyses done—eg analyses of subgroups and interactions, and sensitivity analyses | NA |
| Discussion | | | |
| Key results | 18 | Summarise key results with reference to study objectives | 9 |
| Limitations | 19 | Discuss limitations of the study, taking into account sources of potential bias or imprecision. Discuss both direction and magnitude of any potential bias | 11-12 |
| Interpretation | 20 | Give a cautious overall interpretation of results considering objectives, limitations, multiplicity of analyses, results from similar studies, and other relevant evidence | 9-13 |
| Generalisability | 21 | Discuss the generalisability (external validity) of the study results | 11 |
| Other information | | | |
| Funding | 22 | Give the source of funding and the role of the funders for the present study and, if applicable, for the original study on which the present article is based | 1 |

*Give information separately for cases and controls in case-control studies and, if applicable, for exposed and unexposed groups in cohort and cross-sectional studies.

**Note:** An Explanation and Elaboration article discusses each checklist item and gives methodological background and published examples of transparent reporting. The STROBE checklist is best used in conjunction with this article (freely available on the Web sites of PLoS Medicine at http://www.plosmedicine.org/, Annals of Internal Medicine at http://www.annals.org/, and Epidemiology at http://www.epidem.com/). Information on the STROBE Initiative is available at [www.strobe-statement.org](http://www.strobe-statement.org). Page numbers reflect manuscript submission before final formatting for publication.

**Supplemental Figure 1.** Number and Percent of Advanced Bionics Device Complications from 2016 to 2024
